# Optimization and Appraisal of Nintedanib-Loaded Mixed Polymeric Micelles as a Potential Nanovector for Non-Invasive Pulmonary Fibrosis Mitigation

**DOI:** 10.3390/ph17101275

**Published:** 2024-09-26

**Authors:** Heba M. Aboud, Shahira F. El Menshawe, Nada H. Mohammed, Alaa S. Tulbah, Adel A. Ali

**Affiliations:** 1 Department of Pharmaceutics and Industrial Pharmacy, Faculty of Pharmacy, Beni-Suef University, Beni-Suef 62514, Egypt; shahira.elmenshawe@yahoo.com (S.F.E.M.); adel.ali@pharm.bsu.edu.eg (A.A.A.); 2 Department of Pharmaceutics and Pharmaceutical Technology, Faculty of Pharmacy, Deraya University, Minia 61768, Egypt; 3 Pharmaceutical Sciences Department, College of Pharmacy, Umm Al-Qura University, Makkah 24382, Saudi Arabia; astulbah@uqu.edu.sa

**Keywords:** pulmonary fibrosis, non-invasive targeting, nintedanib, polymeric mixed micelles, statistical optimization, pharmacokinetics

## Abstract

Background/Objectives: Nintedanib (NTD), a triple tyrosine kinase receptor inhibitor, is the recommended first-line tackling option for idiopathic pulmonary fibrosis (IPF). Nevertheless, the adequacy of NTD is curtailed by issues associated with its low solubility, first-pass effect, poor bioavailability, and liver toxicity. The objective of our work was to develop a non-invasive intratracheal (i.t.) nanoparadigm based on NTD-loaded polymeric mixed micelles (NTD-PMMs) that can effectively treat IPF by sustaining the release of NTD, and snowballing its bioavailability, solubility, and efficacy. Methods: Design-Expert^®^ software was used to optimize various NTD-PMMs formulations via Box–Behnken design adopting the thin-film hydration technique. The optimum formulation was chosen and in vivo tested in a rat model to explore its comparative bioavailability and toxicity. Results: The formulation composition with 309.217 mg of Soluplus, 150 mg of Tween 80, and 40 mg of sodium deoxycholate was found to fulfill the requisites of an optimum NTD-PMMs formulation. The optimum NTD-PMMs formulation divulged 90.26% entrapment efficiency with a surface charge of −14.72 mV and a nanoscale diameter of 61.36 nm. Also, it substantially sustained the release of NTD by 66.84% after 24 h and manifested a pronounced stability. In vivo histopathology investigations verified the safety of NTD-PMMs delivered intratracheally. Moreover, pharmacokinetic analyses disclosed accentuated relative bioavailability of the optimized NTD-PMMs by 2.4- and 3.82-fold as compared with both the i.t. and oral crude NTD suspensions, respectively. Conclusions: Overall, the current results elicited the potential of PMMs to serve as a promising pulmonary nanovector for the targeted delivery of NTD.

## 1. Introduction

Pulmonary disorders have aroused a significant public health concern pursuant to their unfavorable prognosis and ineffective treatment strategies in the earlier decade [1]. An exacerbation of lung disorders was triggered during and after the COVID-19 pandemic, resulting in significant difficulties for healthcare professionals. Idiopathic pulmonary fibrosis (IPF) is an uncommon and progressive chronic lung disease characterized by unknown causes, leading to mental and physical distresses [2]. A significant fraction of the global yearly mortality rate is attributable to fibrosis. According to fibrosis epidemiology, there are 0.09–1.30 acquired incidences and 0.33–4.51 existing instances for every 10,000 individuals [3].

Acute respiratory distress syndrome brought on by COVID-19 has caused an increase in IPF patients recently [1,4]. Pathophysiological inflammation induces activation of lung macrophages, which in turn stimulates transforming growth factor-β1, leading to the trans-differentiation of fibroblasts into myofibroblasts [5]. This results in the uncontrolled creation of extracellular matrix and collagen, causing lung fibrosis [6]. IPF results in compromised gas exchange in the lung tissues, resulting in a median lifespan of 2 to 3 years if not treated [1,7]. Currently, there is no enduring remedy for IPF, and lung transplantation remains the only alternative for people with IPF [8].

Collagen synthesis inhibitors, glucocorticoids, immunosuppressants, cytokine inhibitors, tyrosine kinase receptor inhibitors, endothelin antagonists, and anticoagulants are the most common clinically prescribed therapies for IPF patients. Actually, nintedanib (NTD), a triple tyrosine kinase receptor inhibitor, is the recommended first line of treatment for IPF. NTD selectively engages to the ATP-binding sites of platelet-derived growth factor receptors, vascular endothelial growth factor receptors, and fibroblast growth factor receptors [9]. In addition, NTD has received increasing attention due to its immunoregulatory effects [10]. A recent study confirmed that NTD reduced intermediate and advanced pulmonary fibrosis by inhibiting the polarization of M2 macrophages [10]. NTD is a safe medication and slows IPF disease progression by reducing the rate of decline in forced vital capacity and its mortality and acute exacerbations [11]. NTD is classified as a BCS II biopharmaceutical and has a very low oral bioavailability of 4.7% because of its poor aqueous solubility [12]. Furthermore, NTD has been linked to serious adverse effects that need constant monitoring, including liver toxicity, gastrointestinal perforation, and atherosclerosis, which comprises bleeding, arterial thrombosis, and myocardial infarction [13]. As a result, the medication is recommended for inhaled delivery owing to its unique merits, which encompass low systemic side effects, quick clinical response, high local concentration, bypassing first-pass hepatic metabolism, large alveolar surface area, and thin blood–alveolar barrier [14,15]. In addition, as compared to other routes such as parenteral administration, the non-invasive and painless nature of this route ensures that patients are more likely to comply with the treatment [15]. Consequently, patients have an improved quality of life and achieve more favorable treatment outcomes [16,17].

In this regard, nanotechnology has provided an appropriate environment for the development of innovative respirable drug delivery methods that may ameliorate IPF treatment. To fabricate a safe and efficient inhaled drug, it is important to select an appropriate nano-cargo for its delivery and produce a formulation that can be easily inhaled together with the active moiety. These carriers at the nanoscale ranges have the potential to enhance the effectiveness of medications by facilitating their deposition in the deep regions of the lungs [18]. Numerous researchers have investigated the pulmonary delivery of NTD using nanosized carriers, such as liposomes [10], nanosuspension [19], PLGA nanoparticles [20,21], niosomes [22], inhalable powders, and lipid polymer hybrid nanoparticles [16,23].

Among the diverse nano-cargos, polymeric mixed micelles (PMMs) confer an appealing platform for developing innovative drug delivery systems for pulmonary distribution. In particular, PMMs are nanoscale carriers composed of amphiphilic polymers that have the ability to spontaneously form aggregates in water when their concentration is beyond the critical micelle concentration (CMC) [24]. PMMs are composed of a hydrophilic corona around a hydrophobic core. Lipophilic pharmaceuticals can be encapsulated by the latter, whereas the former promotes colloidal stability in the nano-cargo [25]. Furthermore, these nano-cargos demonstrate enhanced stability and solubility to hydrophobic medicines, along with various in vitro and in vivo benefits [26]. Because of their dimensions, they are sufficiently large to avoid early excretion by glomerular filtration and yet small enough to traverse certain blood vessels [27]. In addition, they can improve the drug-loaded micelles’ cellular absorption, lengthen the drug’s average time in circulation, boost bioavailability, decrease the dosage required to achieve the desired effect, and potentially reduce non-specific organ toxicity by delivering the drug to the right tissues at the right time [24,25,26,27].

Recently, Soluplus, an innovative biomaterial, has been effectively exploited in the creation of PMMs [28]. This graft copolymer is distinguished by its exceptional solubilizing traits for pharmaceuticals that are not readily soluble in water. It is composed of poly (vinyl caprolactam), poly (vinyl acetate), and poly (ethylene glycol) [29,30]. In addition, Soluplus micelles have excellent stability when diluted, which is attributed to their low CMC [24]. To our best knowledge, there has been no research investigating the possibility of PMMs for pulmonary targeting of NTD.

Hence, the aim of this work was to develop PMMs containing NTD for its pulmonary targeting in order to ensure efficient administration of NTD to the lungs. The Box–Behnken statistical design was used to get a concentrated and stable formulation of NTD with a notable nanosize and well-defined physicochemical properties. Furthermore, a comprehensive study was performed to assess the irritating effects of the optimized NTD-PMMs formulation on the lungs of rats using in vivo histopathology. Finally, the pharmacokinetic characteristics of NTD-PMMs nanosuspension following intratracheal (i.t.) administration in male Wistar rats were compared to those of i.t. and oral crude NTD suspensions.

## 2. Results and Discussion

PMMs have a variety of intriguing attributes as nanovectors, such as their alterable composition, diminutive size, feasible assembly, and little toxicity to cells. Micelles are most likely created by a reversible process of aggregation, where it is essential to maintain the appropriate HLB (hydrophilic-lipophilic balance) of the nanosystem to guarantee their stability. PMMs are less likely to form in hydrophobic systems, whereas hydrophilic systems become less stable. Hence, the careful selection of polymer amounts is essential in order to attain both kinetic and thermodynamic stability in nanodispersions [31]. We endeavored to develop NTD-PMMs that were transparent, small-sized, and stable by employing Soluplus/Tween 80/SDC in this investigation, without the necessity of external energy processing. These PMMs could efficiently improve the solubility of NTD in water.

### 2.1. Experimental Design

Experimental design is used as a reliable method to reduce process variance while also providing other benefits such as accuracy, precision, and prognosis [31]. The Box–Behnken design (BBD) was used to optimize and analyze the main, joint, and quadratic effects of the multivariable process. Based on rotatable or roughly rotatable second-order designs, it is an incomplete three-factor, three-level factorial design. By using the BBD, the sample size is limited to a number that is necessary for the assessment of the coefficients in a second-degree least square, nearly polynomial form. One important characteristic of the BBD is that it nullifies experimental trials conducted under harsh conditions and does not concurrently implicate combinations in which all variables are at their lowest or highest values [32]. The BBD resulted in 15 empirical runs for creating NTD-PMMs with triple checkpoints, as specified in Table 1.

**Table 1:** Levels of independent variables and results of dependent variables for NTD-PMMs formulations using the Box–Behnken design.

**Independent Factors**					**Levels**		
				−1		0	1
**X_1_: Soluplus concentration (mg)**				200		300	400
**X_2_: Tween 80 concentration (mg)**				150		200	250
**X_3_: SDC concentration (mg)**				0		20	40
**Run**	**X_1_**	**X_2_**	**X_3_**	**PS (Y_1_, nm)**	**EE (Y_2_, %)**	**ZP (Y_3_, mV)**	**CR (Y_4_, %)**
R1	0	1	1	77.93 ± 3.49	76.59 ± 0.49	–12.87 ± 0.25	40.25 ± 0.70
R2	1	0	1	133.03 ± 4.66	75.44 ± 0.74	–17.63 ± 1.05	21.24 ± 1.76
R3 *	0	0	0	59.53 ± 3.69	85.19 ± 1.86	–10.53 ± 2.22	41.70 ± 3.05
R4	1	0	−1	122.03 ± 2.95	82.73 ± 0.43	–14.40 ± 0.87	32.46 ± 2.08
R5	0	1	−1	71.47 ± 2.29	82.09 ± 1.17	–8.40 ± 0.26	45.06 ± 0.98
R6	−1	1	0	102.87 ± 3.58	84.54 ± 0.77	–4.03 ± 0.57	35.87 ± 1.94
R7 *	0	0	0	62.60 ± 2.16	85.17 ± 1.36	–11.40 ± 0.75	49.62 ± 8.21
R8	1	1	0	144.00 ± 1.73	74.21 ± 2.56	–15.63 ± 0.85	24.72 ± 1.69
R9	−1	0	−1	87.47 ± 2.15	93.51 ± 0.21	–3.97 ± 0.64	49.28 ± 2.80
R10	1	−1	0	113.27 ± 2.25	83.31 ± 0.71	–17.77 ± 1.10	38.78 ± 1.14
R11	−1	0	1	106.33 ± 3.31	86.47 ± 0.47	–6.80 ± 0.69	41.69 ± 2.65
R12	0	−1	−1	45.83 ± 3.91	94.30 ± 1.28	–9.33 ± 0.21	77.14 ± 2.09
R13 *	0	0	0	61.67 ± 2.08	85.18 ± 1.58	–11.40 ± 2.13	49.84 ± 8.45
R14	0	−1	1	59.40 ± 3.15	87.41 ± 1.11	–13.60 ± 0.36	68.39 ± 1.88
R15	−1	−1	0	94.80 ± 2.55	96.14 ± 1.21	–5.70 ± 0.61	59.93 ± 2.71

NTD: nintedanib; PMMs: polymeric mixed micelles; SDC: sodium deoxycholate; PS: particle size; EE: entrapment efficiency; ZP: zeta potential; CR: cumulative % release after 24 h. Data denote mean *±* SD (*n* = 3). * Specifies the center point of the design.

As marked in Table 2, the results obtained from the design analysis were presented. The results indicated a strong correlation between the adjusted and predicted R^2^ values. All the responses in Table 2 had a precision of more than 4. The statistical investigation was conducted using multiple regression analysis in the Design-Expert^®^ program (Version 12.0.3.0, Stat Ease Inc., Minneapolis, MN, USA). The model that exhibited the highest level of significance was also used to represent each response variable. Regression equations were used to evaluate the magnitude and mathematical sign of each independent variable. A positive sign denotes a synergistic influence, whereas a negative symbol signifies an antagonistic effect. Table 2 displays a compilation of mathematical equations represented by coded values. These equations depict the essential connections between the independent variables and the corresponding response variables.

**Figure 1:**
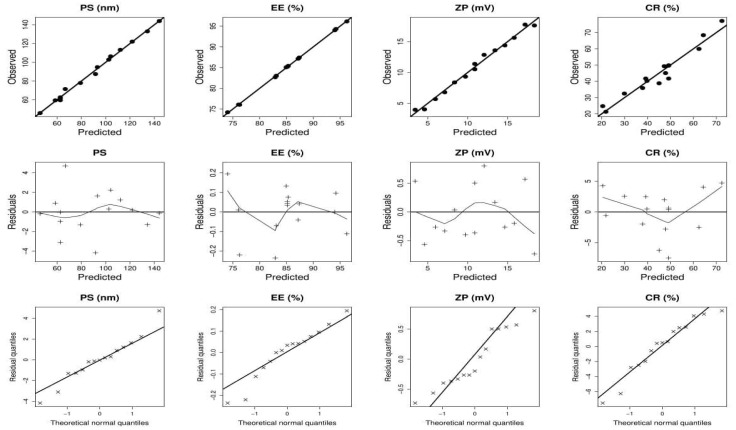
Model diagnostics graphs for NTD-PMMs dependent variables produced by the R software. PS: particle size; EE: entrapment efficiency; ZP: zeta potential; CR: cumulative % release after 24 h.

**Figure 2:**
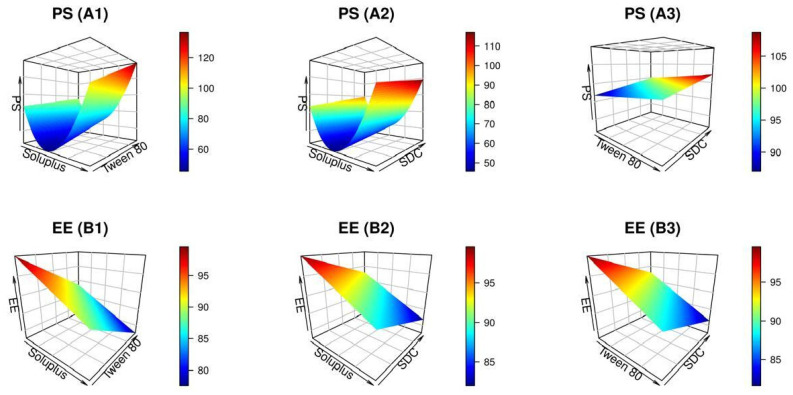
Plots in three dimensions produced by R software demonstrating how the levels of independent variables impact the particle size (PS) (**A1**–**A3**) and entrapment efficiency (EE) (**B1**–**B3**) of the NTD-PMMs.

**Figure 3:**
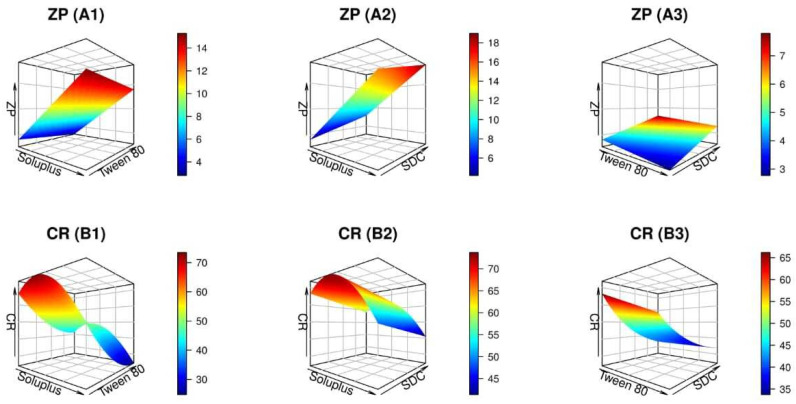
Plots in three dimensions produced by R software demonstrating how the levels of independent variables impact the zeta potential (ZP) (**A1**–**A3**) and cumulative release (CR) after 24 h (**B1**–**B3**) of the NTD-PMMs.

Table 1 denotes the results of the response variables obtained from 15 experimental trials conducted with varying levels of independent factors. The wide range of dependent factors suggests that the characteristics of PMMs may be significantly influenced by the values of independent variables. The models accurately explained the observed variation, as shown by the minimal lack of fit (Table 2). Furthermore, the ANOVA analysis revealed that all independent factors had a significant impact on the response variables. Moreover, the diagnostic diagrams shown in Figure 1 provided additional evidence supporting the appropriateness of the fitted models. The relationship between the causal factors and the response variables is shown graphically in Figure 2 and Figure 3. In each 3D graphic, the third variable remains constant at its center value, while the impact of the other two independent variables is shown.

**Table 2:** ANOVA results for the response variables using Design-Expert^®^ Software.

Source	PS (Y_1_)		EE (Y_2_)		ZP (Y_3_)		CR (Y_4_)	
	F	*p*	F	*p*	F	*p*	F	*p*
Model	432.95	<0.0001	404.06	<0.0001	190.18	<0.0001	55.73	<0.0001
X_1_: Soluplus (mg)	563.37	<0.0001	527.21	<0.0001	513.21	<0.0001	119.84	<0.0001
X_2_: Tween 80 (mg)	265.45	<0.0001	498.53	<0.0001	4.91	0.0322	180.87	<0.0001
X_3_: SDC (mg)	96.02	<0.0001	186.43	<0.0001	52.41	<0.0001	23.67	<0.0001
Lack of Fit	1.76	0.1755	1.13	0.3701	0.6757	0.7247	2.80	0.0556
Model	Reduced Quadratic		Linear		Linear		Reduced Quadratic	
R^2^	0.9911		0.9673		0.9539		0.9348	
Adjusted R^2^	0.9888		0.9649		0.9505		0.9180	
Predicted R^2^	0.9850		0.9610		0.9474		0.9052	
Adequate precision	67.7579		62.0143		50.0291		26.5304	
Standard deviation	3.12		1.20		1.00		0.3190	
%CV	3.48		1.41		9.19		4.82	
$PS=61.27+15.11.X_{1}+10.37.X_{2}+6.24.X_{3}+5.67.\;{X_{1}X_{2}-1.97.\;X_{1}X_{3}-1.78.\;X_{2}X_{3}+50.51.X}_{1}^{2}+1.95{.X}_{2}^{2}+0.4375{.X}_{3}^{2}$								
$EE=84.82-5.62.X_{1}-5.47.X_{2}-3.34.X_{3}$								
$ZP=10.90+5.62.X_{1}-0.6833.X_{2}+1.85.X_{3}$								
$Sqrt\left(CR\right)=6.84-0.7128.X_{1}-0.8756.X_{2}-0.3168.X_{3}+0.1237.\;{X_{1}X_{2}-0.1314.\;X_{1}X_{3}-0.0362.\;X_{2}X_{3}-1.10.X}_{1}^{2}+0.4866{.X}_{2}^{2}+0.1991{.X}_{3}^{2}$								

NTD: nintedanib; PMMS: polymeric mixed micelles; PS: particle size; EE: entrapment efficiency; ZP: zeta potential; CR: cumulative % release after 24 h; CV: coefficient of variance; F: Fisher’s ratio; *P*: probability value.

#### 2.1.1. Effect of Independent Variables on Particle Size (PS)

Actually, micelles with hydrophilic corona are much smaller in size and have a longer in vivo circulation period. Such nano-cargo may monitor the medication administration rate, prolong the therapeutic effect duration, and boost the drug targeting capability to particular tissues [31]. Table 1 and Figure 2(A1–A3) demonstrate that the size of mixed micellar formulations varied between 45.83 ± 3.91 and 144.00 ± 1.73 nm. The increased diffusion mobility within this range renders it suitable for improved cellular absorption and efficient distribution of the drug into the lungs [33]. In addition, ANOVA results for the PS data indicated that the provided dataset is well-suited to the reduced quadratic model. All three independent factors had a substantial influence on the monitored response as shown in Figure 2(A1–A3), with *p* < 0.05.

Figure 2(A1–A3) manifests that NTD-PMMs at lower or higher Soluplus or Tween 80 levels had considerably bigger average sizes than at intermediate concentrations (*p* < 0.05). This finding may be interpreted by the notion that Soluplus enhances the solubility and dispersion of hydrophobic medications, which lowers the PS of mixed micelles. Its amphiphilic trait enables it to interact with both hydrophobic and hydrophilic moieties, therefore stabilizing smaller micelles and impeding their aggregation. On the other hand, upraising the concentration of Soluplus triggered a significant increment in the size of the micelles. This could be owed to the expansion of the outer corona of the micelles and/or an increase in the hydrophilicity of the nanosystem. As a result, the NTD-PMMs became unstable and started to aggregate. Moreover, a higher concentration of Soluplus might substantially elevate the viscosity of the solution, hindering the efficient dispersion of micelles and resulting in the creation of bigger ones. Pignatello et al. also achieved comparable results when they developed systems of Soluplus polymeric nanomicelles to ameliorate the solubility of BCS-class II medications [34].

Taken together, Figure 2(A1,A3) illustrates that the initial elevation of Tween 80 levels provoked the generation of tiny micelles. Tween 80 has the capacity to reduce the PS of mixed micelles by improving the solubility of NTD and strengthening the overall stability of the micelles. The amphiphilic nature of Tween 80 enables it to be efficiently incorporated into the micelle structure, hence subsiding interfacial tension. Conversely, NTD-PMMs could become unstable and aggregate if the concentration of Tween 80 was raised any further; this would be due to the swelling of the micelle’s outer shell, which in turn would be caused by the PEG chains of Tween 80. Such data are similar to that declared by an earlier art on mixed micelles systems encapsulating valsartan [35].

Of note, it was elicited that the PS significantly rose in response to the upsurge in SDC levels, as clarified in Figure 2(A2,A3). The exploitation of an anionic SDC in the assembling materials may explain this result by producing micelles with greater negative ZP. Consequently, the PS of the produced polymeric mixed micelles will grow as the concentration of SDC escalates due to the amplified electrostatic repulsion within the micelles’ corona [36]. As well, the steroidal-like structure of the SDC molecule, which makes it bulky, may have caused the PS to swell when the concentration was elevated. These observations are in line with those of Elsharkawy et al. who investigated the fabrication of lecithin-based polymeric micelles as a delivery vehicle for clozapine via intranasal administration [37].

The homogeneity of the micellar nanodispersions was scrutinized utilizing PDI. A zero value implies a monodispersed nanosystem, whilst a value of one signifies greatly polydispersed particulates into the nanosystem [38,39]. In the current art, PDI of NTD-PMMs fluctuated from 0.091 ± 0.021 to 0.231 ± 0.101, denoting a uniform distribution pattern and adequate homogeneity of the tailored nanoformulations.

#### 2.1.2. Effect of Independent Variables on EE

The propensity of the tailored PMMs for trapping high NTD levels is pivotal for their targeted exploitation as an i.t. nanoparadigm for management of pulmonary fibrosis [40]. Therefore, it is crucial to reduce the leakage of ensnared substances. As profiled in Table 1, the range of EE% values spanned from 74.21 ± 2.56 to 96.14 ± 1.21%. All the independent parameters had a substantial impact on NTD retention in the mixed micellar nano-cargo, as recorded in Table 2. Figure 2(B1–B3) demonstrates a 3D surface diagram of the EE data. The ANOVA analysis indicated that the linear model accurately corresponded to the observed EE data.

The results of the current investigation indicated that Soluplus had a significant impact on EE% (*p* < 0.0001). Figure 2(B1,B2) illustrates the inverse proportionality of this relationship, which implies that an upraised Soluplus level was accompanied by a decline in EE%. One possible explanation for the increased EE% at lower Soluplus concentrations is the presence of fewer hydrophilic PEG chains, which makes it easier to incorporate the NTD. Contrarily, the micellar nano-cargo may have become unstable as a result of the augmented hydrophilicity of the system caused by the upsurge in Soluplus concentration.

It is clear from Figure 2(B1,B3) that as the concentration of Tween 80 was raised, the EE% of NTD decreased synchronously. The micellar system may have also become unstable due to the higher concentrations of Tween 80, which reduced the EE%. This instability could have been caused by the larger PEG chains of Tween 80 in the micelles’ outer shell, which exert a repulsive force against the core’s attractive forces. In addition, a decrement in NTD EE% may be ascribed to self-aggregation of Tween 80 molecules at higher concentrations. These results are congruent with former literature reports [31,35].

Likewise, it was observed that the EE% values of NTD were dropped in tandem with the increase in SDC level. The ability of bile salts to integrate perpendicularly into the micellar core is thought to compromise the EE% of the laded medications since it could enhance drug solubility in the aqueous milieu at the same time as the creation of mixed micelles. Our results are in harmony with those of Duan et al. who delineated a diminished loading efficacy of mixed micelles loaded with silybin at higher SDC concentrations [41].

#### 2.1.3. Effect of Independent Variables on ZP

The ZP measurement is used to predict the stability of colloidal dispersions, therefore, it was deemed essential throughout the optimization process [32]. The value of ZP represents the electrostatic repulsion experienced by neighboring particles with identical charges and distributions. As a result, formulations with high ZP (positive or negative) demonstrate stability by lowering floccule formation and particle aggregation. Table 1 and Figure 3(A1–A3) indicate that the ZP values of all the produced PMMs fluctuated from –3.97 ± 0.64 to −17.77 ± 1.10 mV. Based on the current results, the fabricated nanodispersions were negatively-charged, hence, ZP data were interpreted according to the absolute values to abolish misperception [32]. The linear model was determined to be appropriate for analysis based on ANOVA conducted on the observed ZP data. In addition, the performed ANOVA analysis demonstrated statistical significance of the impact of each independent factor on the ZP of PMMs formulations.

The ZP values rose in direct proportion to the increase in Soluplus level, as expected. The reason for this finding is that Soluplus causes the micelles to have a greater negative ZP because of the polyvinyl caprolactam and polyvinyl acetate segments present in its structure. These segments have the ability to introduce negative charges or amplify the total negative charge on the surface of the micelles, thereby increasing electrostatic repulsion and providing stability to the micelles [42].

Contrarily, there was a negative association between the ZP values and levels of Tween 80, as revealed in Figure 3(A1,A3). This phenomenon may be succumbed to the fact that Tween 80, a non-ionic surfactant, can adhere to the surface of micelles that have a negative charge, therefore partly shielding the negative charge. Additionally, the coexistence of (CH2-CH2-O)_n_ within Tweens would generate hydrogen bonding with molecules of water, triggering dropped ZP [35]. This observation is in harmony with that of El Menshawe et al. who reported diminished ZP values with higher Tween 80 levels upon fabrication of ultra-elastic nanovector for celecoxib colorectal targeting [43].

Moreover, as shown in Figure 3(A2,A3), a high SDC content is correlated with an increase in the ZP values of the generated nanomicelles due to the anionic trait of SDC (presence of cholate group in SDC structure) [44]. Such findings run with those published by Abdelbary et al. in an investigation on the assembly of terconazole-based ocular nano-cargo [45].

#### 2.1.4. Effect of Independent Variables on CR

Table 1 compiles the impact of the independent factors on the release of NTD from PMMs, whilst Figure 3(B1–B3) displays the data in a 3D surface plot. According to the data shown in Table 1, the CR% values oscillated between 21.24 ± 1.76 and 77.14 ± 2.09%. Based on the ANOVA analysis, the reduced quadratic model was appropriate for the observed CR data. Moreover, the ANOVA results indicated that the three independent factors had a significant influence on the release of NTD from PMMs, *p* < 0.05.

Figure 3(B1,B2) demonstrates that the initial increment in Soluplus concentrations was associated with heightened rates of NTD release. Such behavior may be explained by the hydrophilic nature of the copolymer, which enhances water permeability and facilitates the diffusion of NTD across the copolymeric matrix. This leads to the formation of additional hydrophilic channels, resulting in accelerated drug release rates [31]. Furthermore, the small dimensions of these nanoreservoirs could result in a higher surface area-to-volume ratio, which expands the surface area exposed to the release environment. On the other hand, sluggish NTD release rates were elicited with further accretion in Soluplus levels, which may be owed to the aggregation of the micellar shells in addition to their comparatively larger micellar size.

The findings depicted in Figure 3(B1,B3) outlined that the ameliorated NTD release rates observed with higher initial concentrations of Tween 80 could be explained by the hydrophilic nature of the PEG chains used in this dispersion. This property allows for snowballed water permeation and drug diffusion through the polymeric matrix, which in turn leads to the formation of extra-hydrophilic channels and, ultimately, prompt drug release rates [35]. Nevertheless, the expansion of the micelle’s outer shell due to aggregates formation may account for the declined NTD release rates observed with higher Tween 80 concentrations.

According to the regression coefficients, a negative correlation between the concentrations of SDC and CR was manifested, which might be attributed to elevated amounts of SDC that provoked PMMs with bigger PS. This, in turn, would confer a smaller surface area for the efflux of NTD and curtail the release rate [32]. Additionally, the present results clarified the dependency of NTD release on micellar size.

### 2.2. Formulation Optimization

**Figure 4:**
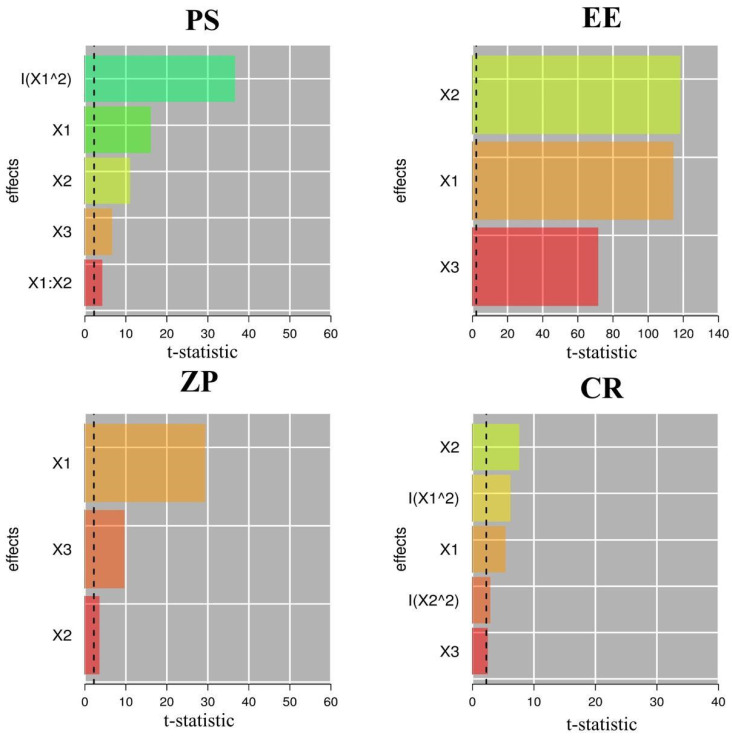
Pareto chart generated by R software representing the standardized impacts of X_1_: Soluplus concentration (mg), X_2_: Tween 80 concentration (mg), and X_3_: SDC concentration (mg). PS: particle size; EE: entrapment efficiency; ZP: zeta potential; CR: cumulative % release after 24 h.

The Design-Expert^®^ program was employed to establish precise criteria for the selection of the most optimal formulation. The settings were monitored for generating PMMs with the minimum PS, highest EE, ZP, and CR. The applied program recommended the development of the optimal NTD-PMMs, which achieved a total desirability value of 0.756. The optimum composition that met the formulation criteria of the PMMs consisted of 309.217 mg of Soluplus, 150 mg of Tween 80, and 40 mg of SDC. The following optimum response variables were noticed: ZP of −14.72 mV, PS of 61.36 nm, EE of 90.26%, and CR of 66.84% (Table 3). The attributes of the optimal PMMs were properly evaluated utilizing the models presented. As listed in Table 3, the predicted error scores for all replicates were less than 5%. The Pareto chart, displayed in Figure 4, accurately demonstrates the consistent impact of formulation parameters on the response variables. The amount of Soluplus (X_1_) had the predominant impact on all response variables.

**Table 3:** The composition of the optimal formulation and its response variables (experimental, predicted, and prediction error).

	**X_1_: Soluplus Concentration (mg)**		**X_2_: Tween 80 Concentration (mg)**		**X_3_: SDC Concentration (mg)**
**Optimal values**	309.217		150		40
**Desirability**	**0.756**				
	**PS (nm)**	**EE%**		**ZP (mV)**	**CR %**
**Predicted**	62.418	86.424		−13.99	64.222
**Experimental**	61.36	90.26		−14.72	66.84
**Prediction error (%) ^£^**	1.72	4.24		4.95	3.92

SDC: sodium deoxycholate; PS: particle size; EE: entrapment efficiency; ZP: zeta potential; CR: cumulative % release after 24 h. ^£^ (Experimental–Predicted)/Experimental × 100.

### 2.3. Characterization of the Optimized Formulation

#### 2.3.1. FTIR Analysis

**Figure 5:**
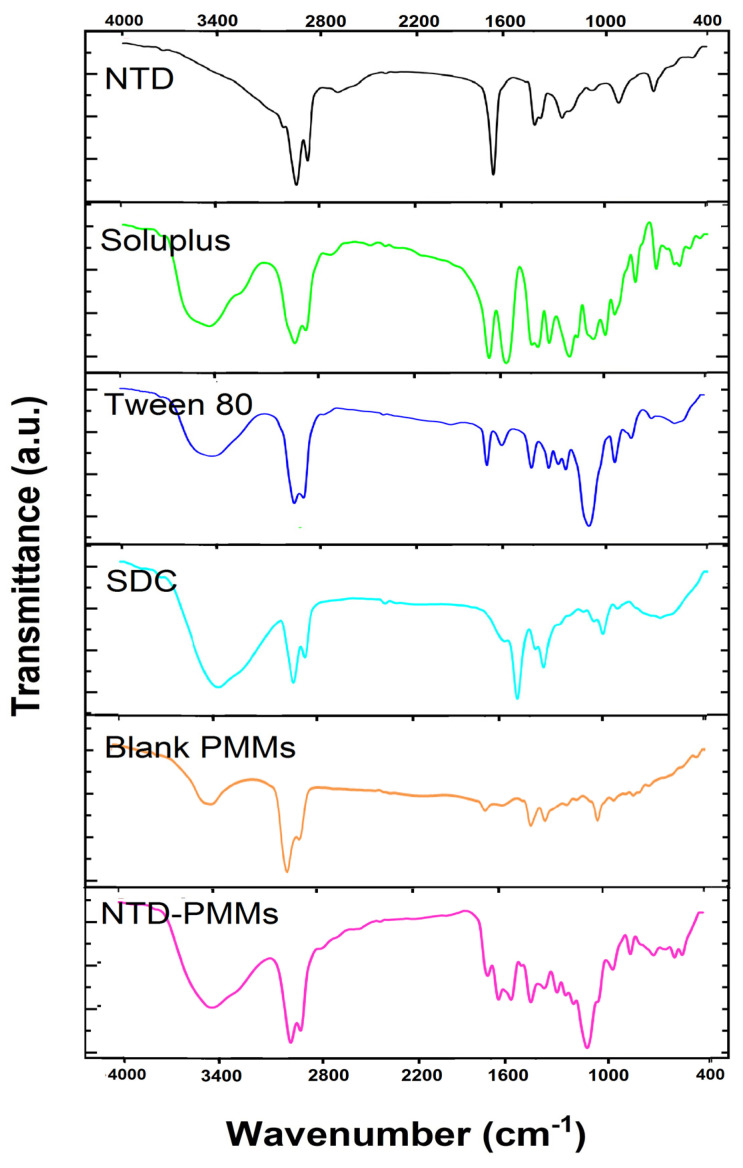
FT-IR spectra of NTD (pure drug), Soluplus, Tween 80, SDC, blank PMMs, and NTD-PMMs (optimal formulation).

The infrared spectra of NTD (pure drug), Soluplus, Tween 80, SDC, and NTD-PMMs (optimal formulation) are presented in Figure 5. The NTD spectrum exhibits distinctive peaks at 2671.84, 2376.86, 2926.96, and 2858.06 cm^−1^ (outlining C-H stretching, CH3), 1457.11 cm^−1^ (signifying the C=O stretch of the amide), 1712.08 cm^−1^ (elucidating the C=O stretch of the ester group), and 1422.14 and 1288.38 cm^−1^ (marking the C-N stretch) [1]. Three further significant peaks were observed at 1106.96, 939.52, and 723.83 cm^−1^. Also, Figure 5 depicts the IR spectra of Soluplus, Tween 80, SDC, blank PMMs, and NTD-PMMs. The IR spectrum of blank PMMs manifested the same characteristic bands for Soluplus, Tween 80, and SDC. Additionally, the IR spectrum of NTD-PMMs displayed the same characteristic bands for NTD, both in terms of position and intensity, which is rather intriguing (Figure 5). In some cases, the intensity might be reduced as a result of dilution in the combination; still, no further bands were noticed. These findings could point out that none of the investigated compounds disclosed any evidence of chemical interactions with NTD [46].

#### 2.3.2. Differential Scanning Calorimetry

**Figure 6:**
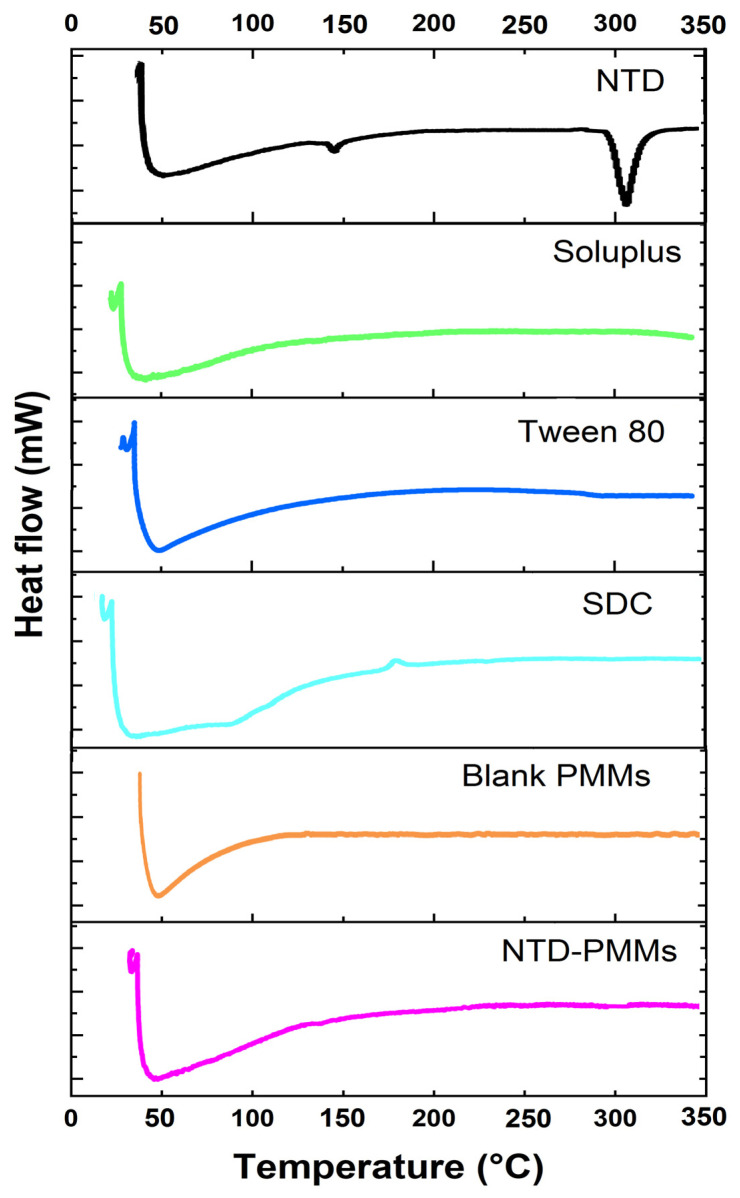
DSC thermograms of NTD (pure drug), Soluplus, Tween 80, SDC, blank PMMS, and NTD-PMMs (optimal formulation).

Using the DSC instrument, thermal tests were conducted to study the effects of drug inclusion and crystallinity level of NTD within the nanocarrier (Figure 6). NTD exhibited sharp endothermic peaks at 143.95 °C (T_start_ = 140.50 °C; T_end_ = 146.37 °C; Heat = 13.72 J/g) and 308.32 °C (T_start_ = 295.26 °C; T_end_ = 316.48 °C; Heat = 53.86 J/g) [4]. Additionally, Figure 6 depicts the DSC thermograms of Soluplus, Tween 80, SDC, blank PMMs, and NTD-PMMS. The DSC thermograms of blank PMMs and NTD-PMMs were coincident, revealing the absence of NTD characteristic peaks in the drug-laden thermogram. Nevertheless, the lack of NTD peaks in the thermogram of NTD-PMMs could emphasize the presence of the medication in an amorphous form, which is expected to boost its solubility and permeability [47].

#### 2.3.3. In Vitro Release

**Figure 7:**
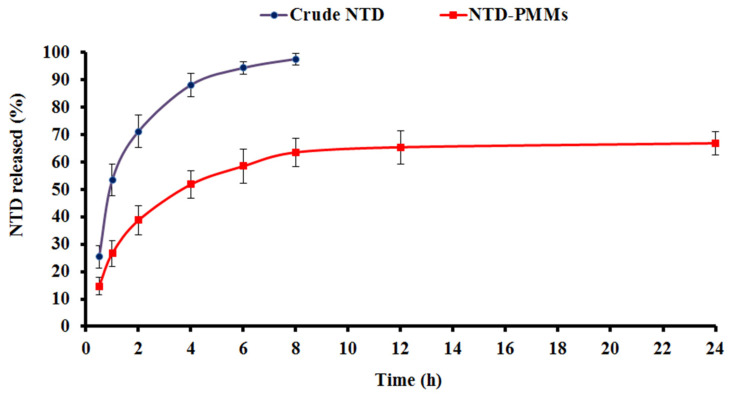
In vitro release profiles of crude nintedanib (NTD) and NTD-PMMs dispersion (optimal formulation) in phosphate buffered saline containing Tween 80 (0.5% *w*/*v*), mean ± SD, n = 3.

The dialysis membrane method was used to assess the in vitro release patterns of the optimum NTD-PMMs formulation and free NTD over a period of 24 h, as represented in Figure 7. The cumulative release of free NTD was 71.17 ± 5.86% after 2 h and upgraded to 7.58 ± 2.16% after a total of 8 h. On the other hand, the total quantity of NTD released from NTD-PMMs was 38.72 ± 5.25% after a 2-h period and 66.84 ± 4.29% after 24 h. This might be due to the medication being encapsulated inside the nano-cargo, which effectively restricts its diffusion into the surrounding environment. The in vitro release profile elicited an initial rapid release during the first 2 h; thereafter, the medication continued to be released steadily from the NTD-PMMs dispersion. The relative impact of the tailored micellar dispersion as a nanoreservoir could highlight the potential cause of the prolonged drug release from NTD-PMMs, which persists after the initial burst release.

#### 2.3.4. Morphological Analysis

**Figure 8:**
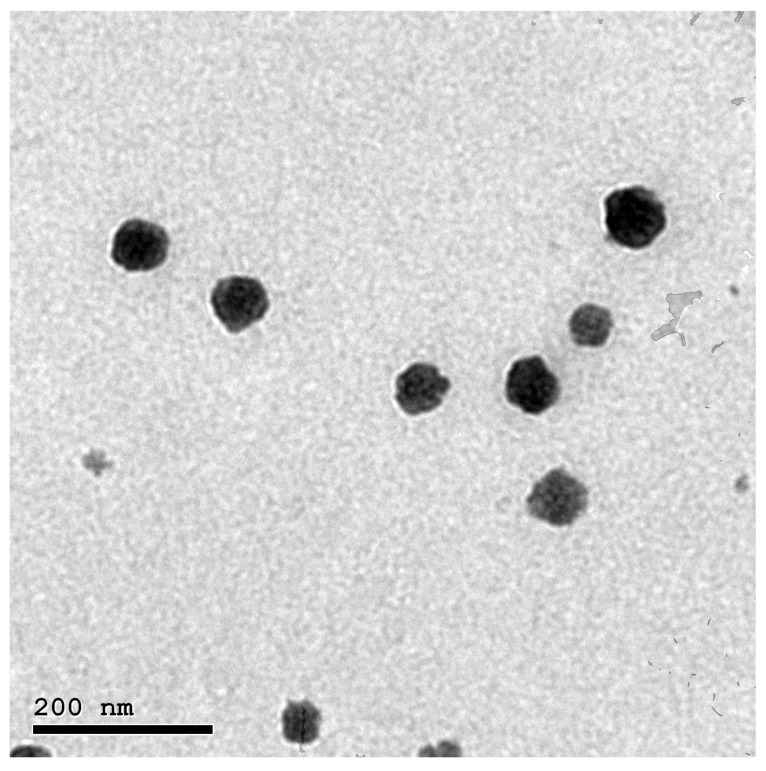
TEM photomicrograph of the optimal NTD-PMMs.

The TEM exploration of the NTD-PMMs morphology demonstrated a spherical shape and uniform distribution without any aggregation. Figure 8 designates well-stained micelles with a suitable arrangement of hydrophobic and hydrophilic segments, which are alternately black and weakly stained. The nanomicelles were also adequately spaced apart and evenly distributed. The analysis of the TEM micrographs also validated the presence of nanosized micelles, which was consistent with the results obtained from DLS measurements.

#### 2.3.5. Short-Term Stability

**Figure 9:**
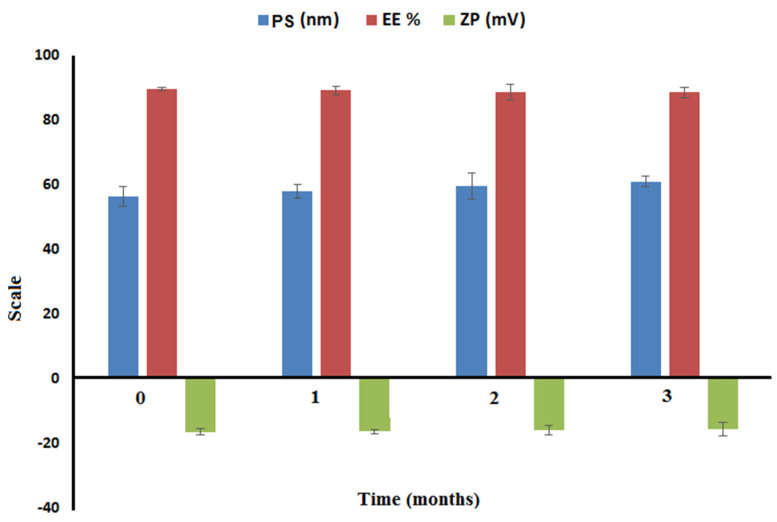
The stability study outcomes after three months of storage for the optimal NTD-PMMs. PS: particle size; EE: entrapment efficiency; ZP: zeta potential.

Values for EE%, PS, and ZP were used to evaluate the physical stability of the optimized NTD-PMMs formulation after three months of storage at 4 °C. There was no aberration or aggregation noted during storage. Figure 9 demonstrates that the EE%, PS, and ZP of the nanoformulation were insignificant to the duration of storage. The high negative ZP of the elaborated optimal NTD-PMMs revealed dispersion stability. The presence of SDC might have contributed to the negative charge on the surface of PMMs, reinforcing their stability in the aqueous phase [44].

### 2.4. In Vivo Studies

#### 2.4.1. Histopathological Analysis

**Figure 10:**
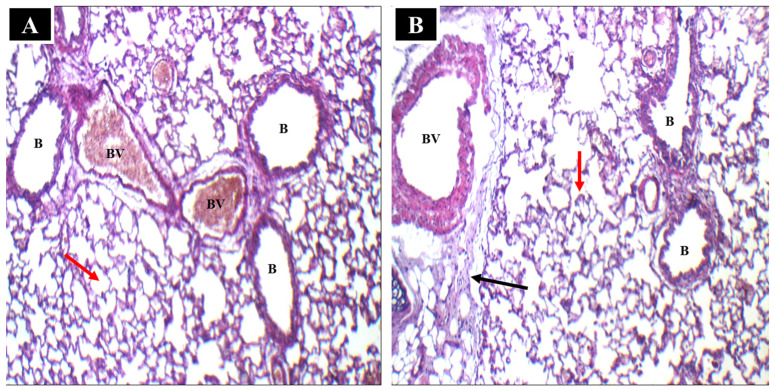
Histopathological section images of (**A**) a rat lung without treatment (control) and (**B**) a rat lung after i.t. administration of NTD-PMMs suspension. (200× H & E). B: bronchioles; BV: blood vessels; red arrow: alveolar walls; black arrow: mild interstitial inflammatory infiltrate.

The lung tissues of rats subjected to optimal i.t. NTD-PMMs were compared with those of normal rats to identify any acute toxicity of the given formulation. The inhalation approach might cause medications to be distributed asymmetrically; hence, the left and right lungs were investigated separately. Figure 10A shows average blood vessels (BV), average alveolar walls (red arrow), and average bronchioles (B). Moreover, there was no epithelial damage, edema, perivascular, or peribronchial inflammation. The lung tissue of the group treated with the optimal NTD-PMMs formulation is depicted in Figure 10B. The lung exhibits average bronchioles (B), average blood vessels (BV), average alveolar walls (red arrow), and mild interstitial inflammatory infiltrate (black arrow). Moreover, neither the left nor the right lung of the animal receiving the i.t. NTD-PMMs formulation revealed any meaningful tissue inflammation or damage intratracheally. Based on the results of this work, the proposed NTD-PMMs formulation was biocompatible and did not generate acute toxicity because of its nano-cargo components.

#### 2.4.2. Pharmacokinetic Study

**Figure 11:**
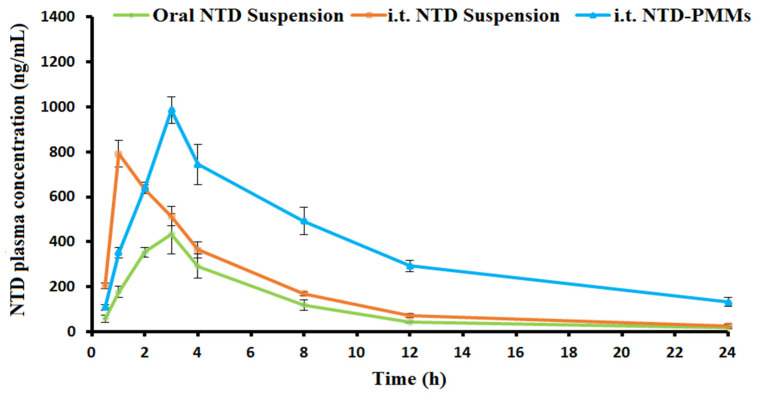
NTD levels in rat plasma following administration of i.t. NTD-PMMs, i.t. NTD suspension and oral NTD suspension.

Male Wistar rats were administered oral NTD suspension, i.t. NTD suspension, and i.t. NTD-PMMs. Subsequently, the plasma concentrations of NTD in the rats were determined. Table 4 displays the corresponding pharmacokinetic characteristics for various formulations, whereas Figure 11 illustrates the average plasma concentration of NTD with time. As outlined in Figure 11, the i.t. administration of NTD-PMMs caused a rapid increase in NTD concentration after 3 h, which was followed by an exponential drop in circulation. This might be the result of the extended liberation of the NTD from the PMMs after administration. The in vitro release profile, shown in Figure 7, further verifies the observed phenomenon. It was shown that the circulation contains measurable amounts of NTD for a duration of 24 h, which indicates the ongoing efflux of NTD from the developed NTD-PMMs.

Based on the current results, the T_max_ values for the NTD suspension were 3 h when taken orally and 1 h when delivered intratracheally. However, in less than 8 h, they were almost eliminated from circulation, as profiled in Figure 11. When oral NTD suspension, i.t. NTD suspension, and i.t. NTD-PMMs were administered, the corresponding AUC_0-∞_ of NTD in plasma were 2729.95 ± 410.11, 4339.16 ± 148.71, and 10,427.60 ± 427.01 ng h/mL. The i.t. NTD-PMMs suspension had a significantly larger AUC_0-∞_ value compared to oral and i.t. NTD suspensions, with nearly 3.82- and 2.40-fold, respectively, *p* < 0.05.

Oral NTD suspension, i.t. NTD suspension, and i.t. NTD-PMMs were delivered, and the C_max_ of NTD was found to be 434.73 ± 76.95, 790.71 ± 43.16, and 986.13 ± 76.46 ng/mL. The C_max_ value was substantially greater after the i.t. administration of NTD-PMMs of about 2.27 and 1.25 times higher than the oral and i.t. NTD suspensions, respectively. In addition, the suspension of NTD-PMMs was shown to have a half-life of 7.94 ± 0.14 h after i.t. administration. In contrast, the half-life values of the NTD suspension were 4.91 ± 0.27 h and 4.65 ± 0.32 h for the oral and i.t. administrations, respectively. The extended half-life of NTD-PMMs demonstrated that NTD was continuously and increasingly absorbed after being administered by i.t. instillation. This suggests that the PMMs reservoir effectively hinders the breakdown of NTD in the lung.

When compared to the oral NTD suspension, the F_rel_ of NTD from the NTD-PMMs suspension was around 381.97%, whilst it was 158.95% for the i.t. NTD suspension. Table 4 clarifies that administering NTD by i.t. instillation, whether in the form of free drug suspension or nanosuspension, significantly enhanced the pharmacokinetic properties in comparison to the oral NTD suspension. This indicates that the desired treatment outcomes for NTD might be successfully achieved by pulmonary delivery. The results of this study elucidated that the i.t. administration of NTD-PMMs boosted the amount of NTD in the bloodstream, as disclosed by the rising plasma drug concentration, snowballed area under the curve, and longer half-life. These observations could mark the accentuated bioavailability of NTD in Wistar rats.

The enhanced bioavailability of NTD when encapsulated in Soluplus-based mixed micelles and delivered via the pulmonary route may be attributable to diverse aspects. The Soluplus mixed micelles could augment the solubility of NTD, a medication that is sparsely soluble in water, hence promoting greater dispersion and absorption in the lung tissues. Additionally, the pulmonary route would circumvent the first-pass metabolism linked to oral administration and enable direct distribution to the site of action, which might greatly minimize drug degradation and improve bioavailability. The micelles further could trigger a regulated release pattern, ensuring a consistent level of NTD in the lungs, thereby enhancing the effectiveness of the treatment. Furthermore, the surfactant aptitude of Soluplus might enhance the permeability of the medication across the pulmonary epithelium, conferring more efficient drug absorption into the systemic circulation. The higher permeability, avoidance of first-pass metabolism, direct distribution, and greater solubility all together would donate the superior pulmonary bioavailability of NTD.

## 3. Materials and Methods

### 3.1. Materials

Nintedanib (NTD) was supplied by Dideu Industries Group Limited (Shaanxi, China). Soluplus, Tween 80, sodium deoxycholate (SDC), acetonitrile (HPLC grade), dialysis membrane (MW cut off: 12 kDa), sodium carboxymethyl cellulose, carbamazepine, formic acid (HPLC grade), and methanol (HPLC grade) were procured from Sigma-Aldrich (St. Louis, MO, USA). Potassium chloride, sodium chloride, disodium hydrogen phosphate, and potassium dihydrogen phosphate were purchased from El-Nasr Pharmaceutical Chemical Company (Cairo, Egypt). The remaining materials enrolled in the study were all analytical laboratory-grade.

### 3.2. Design of Experiments

The Box–Behnken design (BBD) was adopted utilizing Design-Expert^®^ software to assess the impact of different causal variables on the features of NTD-PMMs. A design matrix consisting of fifteen formulations of NTD-PMMs and three causative factors was created, with each factor having three levels. Twelve of the formulations specified the exact middle points of the edges of a three-dimensional (3D) cube, while the remaining formulations, which were repeated three times, represented the precise center of the cube. The opted causative variables were Soluplus (X_1_), Tween 80 (X_2_), and sodium deoxycholate (SDC, X_3_) concentrations (mg). The response variables that were dependent on the experiment were particle size (PS: Y_1_, nm), entrapment efficiency (EE: Y_2_, %), zeta potential (ZP: Y_3_, mV as absolute value), and the percentage of drug released after 24 h (CR: Y_4_, %). The values of the independent variables were determined using the results of previous studies, as shown in Table 1. Table 1 presents a comprehensive summary of the composition of fifteen experimental trials carried out to develop NTD-PMMs. The plot 3D R package in R software (Version 4.2.0, 2022) was used to produce 3D response surface diagrams [43,48]. Then, the optimization was accomplished based on a maximal EE, ZP, and CR and a minimal PS to obtain the formulation with the greatest desirability [32]. 

### 3.3. Preparation of NTD-PMMs

NTD-PMMs nanosuspensions were assembled using the thin-film hydration technique with minor modifications [31,35]. In a round-bottom flask with 3 mL of methanol, Soluplus, Tween 80, NTD (10 mg), and SDC were carefully weighed and dissolved. Vacuum was applied, and the organic solvent was gradually evaporated at 40 °C for 15 min using a rotary evaporator (Stuart rotary evaporator, RE300, Wolf Laboratories, North Yorkshire, UK with Stuart vacuum pump, RE3022C, Wolf Laboratories, North Yorkshire, UK). The process of evaporation was stopped after a thin, clear, and dry film had been developed on the flask walls. To ensure the complete elimination of organic solvent residues, the film was subsequently subjected to vacuum in a desiccator for 2 h. Subsequently, the film was hydrated with 10 mL of phosphate-buffered saline (PBS, pH 7.4). The NTD-PMMs were formed by continuing the hydration process at a temperature of 40 °C for 1 h with a rotation speed of 60 rpm until a transparent micellar solution was obtained. The micellar solution that had been prepared was refrigerated at a temperature of 4 °C overnight until it was subjected to further characterization.

### 3.4. Optimization and Characterization of NTD-PMMs

#### 3.4.1. Micelle Size and ZP Analysis

The average micellar size and surface charge of NTD-PMMs were evaluated by dynamic light scattering (DLS) in a Zetasizer Nano ZS (Malvern Instruments, Malvern, UK). To abolish the problem of multi-scattering, each specimen was diluted before analysis by adding distilled water. The analysis was performed at ambient temperature and with the incident beam directed at a 90° angle. The equilibration period before the analysis was 2 min [31,49].

#### 3.4.2. Entrapment Efficiency of NTD

The EE was indirectly determined by subtracting the free drug left in the aqueous milieu from the total drug incorporated into the formulation (10 mg) after separating the micellar suspension [50]. The micellar suspension was subjected to centrifugation for 1 h at 4 °C and 14,000 rpm using a SIGMA cooling centrifuge (model 3–30 K, Steinheim, Germany). The supernatant was removed, diluted, and analyzed using a Shimadzu UV spectrophotometer (model UV-1601 PC, Tokyo, Japan) to measure the concentration of NTD (non-entrapped) at 389 nm. Prior to the analysis, a calibration curve was created in PBS pH 7.4, covering a range of 2–18 μg/mL (R^2^, 0.9971). The entrapment efficiency of NTD was determined using the following formula [32]:

(1) $$EE\%=\frac{Intial\;amount\;added-amount\;in\;supernatant}{Intial\;amount\;added\;}\times 100$$

#### 3.4.3. Cumulative Release (CR) after 24 h

The drug release processes were carried out utilizing the modified vertical Franz diffusion cells, which had an effective diffusional area of 5 cm^2^, as previously narrated [40]. Prior to the experiment, a dialysis membrane was immersed in PBS pH 7.4 for a duration of 24 h. Afterwards, the membrane was placed between the donor and receptor chambers. The donor chamber was filled with different formulations of NTD-PMMs, with each formulation comprising 3 mg of NTD. The experiment was conducted at a temperature of 37 ± 0.5 °C with continuous agitation at a speed of 100 rpm. The receptor chamber was filled with 70 mL of a PBS solution containing Tween 80 at a concentration of 0.5% *w*/*v* [51]. The samples were collected over a period of 24 h, and the concentration of NTD in them was spectrophotometrically determined at a wavelength of 389 nm. Finally, the amount of NTD released after 24 h was assessed.

### 3.5. Characterization of the Optimized Formulation

#### 3.5.1. Fourier Transform Infrared Spectroscopy (FTIR)

The chemical interactions between NTD and the functional groups of the materials used to fabricate PMMs were examined using FTIR spectroscopy (IR435-U-04, Shimadzu, Kyoto, Japan). The KBr pellet approach was used in the FTIR spectrometer in an inert environment, covering a wavenumber range of 4000–400 cm^−1^. FTIR spectroscopy was used to evaluate the spectrum features of several pure components, including NTD (crude medication), Soluplus, Tween 80, and SDC. In addition, the optimized formulation (NTD-PMMs) and blank PMMs were also examined [50,52].

#### 3.5.2. Differential Scanning Calorimetry (DSC)

The probable interactions among the components were examined by DSC analysis, as previously outlined [49,52]. Each sample, consisting of NTD (crude medication), Soluplus, Tween 80, SDC, the optimized formulation (NTD-PMMs), and blank PMMs, was weighed to get a precise amount ranging from 2 to 6 mg. Afterwards, the samples with added weight were placed in aluminum containers. The aluminum plates were sealed hermetically. The DSC experiment was conducted using a calorimeter (DSC-60, Shimadzu, Kyoto, Japan). The reference sample consisted of an aluminum pan that was devoid of any content. The pan in the DSC chamber was subjected to a temperature range from 25 to 350 °C for both of the sample and reference. The nitrogen gas was consistently flowing at a rate of 20 mL/min, while the temperature was increasing at a steady rate of around 10 °C/min. Furthermore, the baseline was calibrated by utilizing two empty aluminum containers under the same experimental circumstances.

#### 3.5.3. In Vitro Release

The release analyses of crude NTD and the optimum formulation were carried out in accordance with the aforementioned description. The donor chamber was replenished with crude NTD and the optimum NTD-PMMs formulation that contained an equivalent quantity of NTD (3 mg). In order to ensure a consistent volume, 1 mL samples of the receptor milieu were taken at specified time intervals and then replaced with an equal volume of receptor milieu. The gathered samples were filtered, and the overall cumulative release of NTD at certain time intervals was determined using spectrophotometric analysis at a wavelength of 389 nm. A correlation analysis was conducted to determine the relationship between the time and the amount of medication that was released.

#### 3.5.4. Morphological Analysis

The morphological properties of the NTD-PMMs formulation were examined using transmission electron microscopy (TEM) under optimal conditions. A single droplet of the freshly formulated NTD-PMMs dispersion was applied onto a grid covered with carbon and kept at a temperature of 25 °C for a duration of 5 min after a suitable dilution. Afterwards, the grid was treated with a 2% *w*/*v* solution of phosphotungstic acid for negative staining. In order to enhance the uptake of the stain, the grid was allowed to settle, and then a filter paper was used carefully to remove any extra stains. The formulation was examined at 80 kV employing TEM analysis (JEM-1400, Jeol, Tokyo, Japan) [32,53].

#### 3.5.5. Short-Term Stability

The stability of the optimized formulation of NTD-PMMs was assessed by storing it at a temperature of 4 °C for a period of three months, as previously mentioned [31,35]. The characteristics of the optimized NTD-PMMs formulation were evaluated at certain time intervals, namely 0, 30, 60, and 90 days. The samples of NTD-PMMs formulation were gathered and assessed for their visual attributes at the specified time intervals. Furthermore, the mean values of EE, PS, and ZP were computed and then recorded.

### 3.6. In Vivo Studies

Male Wistar rats weighing between 200 and 250 g were utilized in this experiment. The animals were kept in large, spacious wire cages, allowing them to free access to food and water. The cages were situated in chambers inside the animal facility that were carefully regulated for humidity, guaranteeing a stable temperature of 25 ± 2 °C. In addition, the animals were exposed to 12 h of light, followed by 12 h of darkness. Anesthesia was induced in the animals by administering ketamine at a dosage of 12.5 mg/kg and xylazine at a dosage of 1.5 mg/kg intraperitoneally [33]. The optimum NTD-PMMs formulation was applied using the Microsprayer^®^ IA-1C i.t. instillation device (Penn-Century, Philadelphia, PA, USA) [54]. The protocol for the in vivo experiments was approved by the Animal Ethics Committee of Beni-Suef University, adhering to the guidelines outlined in the National Institutes of Health Guide for the Care and Use of Laboratory Animals (approval code: 024–032).

#### 3.6.1. In Vivo Histopathological Analysis

An in vivo histopathology investigation was conducted to investigate the ultrastructural changes in lung tissue that occurred as a result of the administration of NTD-PMMs suspension. Using a random process, six rats were divided into two groups, with three rats in each group (n = 3). Group A was identified as the control group, whereas group B was allocated as the treatment group. The treatment group was administered an optimized NTD-PMMs suspension by i.t. administration for a duration of 14 days. After the experimental procedures were finished, the rats were euthanized. Consequently, their lungs were meticulously extracted and preserved in a formalin solution (10%) for further analysis. The lungs were embedded in paraffin wax blocks and incubated at a temperature of 56 °C for 24 h to facilitate histological examination. The lungs were sectioned into 5 μm-thick slices using a microtome. Subsequently, the excised sections were examined using a light microscope following staining with hematoxylin and eosin (H&E) using an established methodology previously described by Bancroft and Gamble [55]. The segments were captured at several levels of magnification using the LEICA digital camera system (model DFC290HD, Heerbrugg, Switzerland), which was coupled to a light microscope.

#### 3.6.2. Pharmacokinetic Study

For the pharmacokinetic study, nine rats were used, and they were divided into three groups at random. Each group consisted of three animals. About 10 mg/kg of body weight was the recommended dose of NTD for oral and inhaled administration [12]. Group A was given an oral suspension of NTD (2 mg/mL), which was produced in PBS pH 7.4 encompassing 0.5% *w*/*v* sodium carboxymethyl cellulose. Following intraperitoneal anesthesia, animals in groups B and C were administered 200 μL of NTD suspension via the i.t. route, as well as 200 μL of the optimized NTD-PMMs suspension, respectively, with each dose comprising 2 mg of NTD. After administering the doses via the i.t. route, a further step was taken to cleanse the syringe and Microsprayer^®^ tubes. This was done by using 50 μL of a 0.9% saline solution. 500 μL of blood samples were obtained from the retro-orbital venous plexus of each rat at 0.5, 1, 2, 4, 8, 12, and 24 h. The samples were collected into heparin-containing vials to avoid coagulation. After acquiring the samples, the plasma was isolated from them using the process of centrifugation, which was conducted for 15 min at a speed of 3000 rpm. The isolated plasma was combined with 4 mL of acetonitrile and vigorously agitated for 5 min. The denatured protein that had been precipitated was then extracted by a cooling centrifugation process lasting 10 min at a temperature of 4 °C. A vacuum concentrator was employed for the evaporation of the transparent supernatant. After dissolving the resultant residue in the mobile phase, it was then injected into the LC-MS/MS apparatus.

##### Chromatographic Conditions

The LC-MS/MS method was used for quantitative analysis of NTD [56]. The setup included a Shimadzu Prominence (Japan) series LC system with a degasser (DGU-20A3), a Zorbax C18 column (4.6 × 50 mm; 3.5 μm PS), and an auto-sampler (SIL-20 AC). Mobile phase A (0.1% formic acid) and mobile phase B (acetonitrile with 0.1% formic acid) were used in a gradient elution procedure for chromatographic separation. The program was configured as follows: 0–2 min (5–5% B), 2–5 min (5–20% B), 5–8 min (20–30% B), 8–12 min (30–50% B), 12–13 min (50–50% B), and 13–14 min (50–5% B). The flow rate was 0.4 mL/min, and the sample injection volume was 10 μL. Carbamazepine was utilized as the internal standard. The ion discharge was set at a voltage of 5 kV. Electrospray ionization (ESI) in positive mode was run in the mass spectrometer. NTD was measured using the multiple reaction monitoring mode (m/z 540.3 → 113.15) and an internal standard (m/z 237.1 → 194.25).

##### Data Analysis

The pharmacokinetic parameters were calculated using non-compartmental analysis using the PK solver, an additional software tool integrated into Microsoft Excel [35]. The greatest concentration accomplished during the analysis was recorded as the maximum detected concentration (C_max_), measured in ng/mL. Furthermore, the duration needed to reach the highest concentration, referred to as T_max_ (h), was calculated. The integral of the plasma concentration-time curve was determined by using the trapezoidal rule up to the most recent recorded time point. The AUC_0–∞_ was calculated by dividing the concentration at the last time point by the elimination rate constant, and this value was then used to compute the residual area. With respect to an oral suspension that functions as a reference, the percentage relative bioavailability (*F_rel_*) of the two *i.t. formulations* can be evaluated as follows [33]:

$$F_{rel}=\frac{{AUC}_{0-\infty }(i.t.\;formulation)}{{AUC}_{0-\infty }(oral\;formulation)\;}\times 100\;$$

### 3.7. Statistical Analysis

The experiments were performed three times, and the results were reported as the average values ± SD. The study used a one-way ANOVA and a Tukey post hoc test to evaluate the significance of the variations among the groups. A *p* value beneath 0.05 designated a statistical significance.

## 4. Conclusions

In the present work, novel PMMs were refined as a feasible nanovector for entrapping the sparsely-soluble anti-fibrotic drug, NTD, for its pulmonary targeting employing Box–Behnken design. The optimum formulation was successfully elaborated, comprising 309.217/150/40 mg of Soluplus/Tween 80/SDC and elicited appropriate micellar size, higher EE%, sustained release pattern, and reinforced kinetic/thermodynamic stability. The histopathological investigations verified the non-irritant trait of the employed nano-cargo in the studied rats. The pharmacokinetic analyses manifested extended MRT and T_1/2_ alongside marked accentuated bioavailability of the optimal NTD-PMMs formulation versus both oral and i.t. crude NTD suspensions. Thus, the engineered i.t. NTD-PMMs could confer a tolerable, non-invasive, and competent nanoparadigm for IPF tackling.

## Figures and Tables

**Table 4:** The plasma pharmacokinetic parameters of NTD after administration of i.t. NTD-PMMs, i.t. NTD suspension, and oral NTD suspension in Wistar rats.

Pharmacokinetic Parameter	Formulation		
	Oral NTD Suspension	i.t. NTD Suspension	i.t. NTD-PMMs
C_max_ (ng/mL)	434.73 ± 76.95	790.71 ± 43.16 ^a^	986.13 ± 76.46 ^a,b^
T_max_ (h)	3.00 ± 0.00	1.00 ± 0.00 ^a^	3.00 ± 0.00 ^b^
MRT (h)	7.16 ± 0.39	6.60 ± 0.66	12.33 ± 0.35 ^a,b^
K_e_ (1/h)	0.1411 ± 0.0078	0.1490 ± 0.01035	0.0873 ± 0.0016 ^a,b^
t_1/2_ (h)	4.91 ± 0.27	4.65 ± 0.32	7.94 ± 0.14 ^a,b^
AUC_0–t_ (ng h/mL)	2605.12 ± 436.84	4160.18 ± 112.60 ^a^	8912.11 ± 332.49 ^a,b^
AUC _0–∞_ (ng h/mL)	2729.95 ± 410.11	4339.16 ± 148.71 ^a^	10,427.60 ± 427.01 ^a,b^
F_rel_	---------	158.95	381.97 ^b^

NTD: nintedanib; PMMs: polymeric mixed micelles; i.t.: intratracheal. Listed data are mean values *±* SD (*n* = 3). Utilizing one-way ANOVA followed by Tukey’s post hoc test. ^a^ *p* < 0.05 versus oral NTD suspension, ^b^ *p* < 0.05 versus i.t. NTD suspension.

## Data Availability

All proceeded data in this work are incorporated into the article.
